# Hourglass-shaped grafts are superior to conventional grafts for restoring knee stability and graft force at knee flexion angle of 30° following anterior cruciate ligament reconstruction: A finite element analysis

**DOI:** 10.3389/fbioe.2022.967411

**Published:** 2022-12-19

**Authors:** Huizhi Wang, Chaohua Fang, Mingzhu Tao, Qinyi Shi, Kaixin He, Cheng-Kung Cheng

**Affiliations:** ^1^ Engineering Research Center for Digital Medicine of the Ministry of Education, School of Biomedical Engineering, Shanghai Jiao Tong University, Shanghai, China.; ^2^ Department of Joint Surgery, The 6th Hospital of Ningbo, Ningbo, Zhejiang, China

**Keywords:** ACL reconstruction, graft geometry, graft force, notch impingement, knee stability

## Abstract

**Background:** Anterior cruciate ligament reconstruction (ACLR) using a generally columnar graft is considered the gold standard for treating anterior cruciate ligament ruptures, but such grafts cannot replicate the geometry and mechanical properties of the native anterior cruciate ligament.

**Purpose:** To evaluate the effectiveness of an innovative hourglass-shaped graft versus a traditional columnar graft for restoring joint stability and graft force, while avoiding notch impingement following anterior cruciate ligament reconstruction.

**Methods:** Finite element models of a human knee were developed to simulate ① An intact state, ② anterior cruciate ligament reconstruction using columnar grafts with different diameters (7.5–12 mm in 0.5 mm increments), ③ anterior cruciate ligament reconstruction using columnar grafts with different Young’s moduli (129.4, 168.0 and 362.2 MPa) and ④ anterior cruciate ligament reconstruction using hourglass-shaped grafts with different Young’s moduli. The knee model was flexed to 30° and loaded with an anterior tibial load of 103 N, internal tibial moment of 7.5 Nm, and valgus tibial moment of 6.9 Nm. The risk of notch impingement, knee stability and graft forces were compared among the different groups.

**Results:** This study found that columnar grafts could not simultaneously restore knee stability in different degree of freedoms (DOFs) and graft force to a level similar to that of the intact knee. The anterior tibial translation and graft force were restored to a near-normal condition when the internal tibial rotation was over-restrained and valgus tibial rotation was lax. A graft diameter of at least 10 mm was needed to restore knee stability and graft force to physiological levels, but such large grafts were found to be at high risk of notch impingement. In contrast, the hourglass-shaped graft was able to simultaneously restore both knee stability and graft force at knee flexion of 30° while also having a much lower risk of impingement.

**Conclusion:** Under knee flexion angle of 30°, an hourglass-shaped graft was better able to restore joint stability and graft force to a near-physiological level than columnar grafts, while also reducing the risk of notch impingement.

## 1 Introduction

The anterior cruciate ligament (ACL) is important for normal functionality and movement of the knee joint. However, given the substantial multi-directional loads placed on the ACL, the ligament is prone to injury, particularly from non-contact athletic activities involving side-step cutting, heavy landing and rapid deceleration with the knee near full extension (Hashemi et al., 2007). In cases of ACL rupture when conservative non-invasive treatment is not viable, reconstruction surgery is considered the gold standard treatment. Replacing the ruptured ligament with a graft is expected to restore normal joint kinematics and function. Commonly used ACL grafts include autologous tendons such as the hamstring tendon and the patellar tendon, allogeneic tendons and artificial grafts (Wang et al., 2020a). Different positions, shapes and quantities of bone tunnels have also been used to achieve anatomic and non-anatomic reconstructions of the bone insertions (Yao et al., 2012; Wang et al., 2020b; Cheng et al., 2021; Cheng et al., 2022). Clinical outcomes have been reported to be good to excellent at 2 years post-surgery (Komzak et al., 2021). However, the long-term outcome of reconstruction is less encouraging, with a high incidence of secondary knee osteoarthritis (OA) at 14 years after surgery (Barenius et al., 2014). Other common complications following ACL reconstruction include impingement of the graft on the femoral intercondylar notch and graft rupture (Orsi et al., 2017; Schmucker et al., 2021).

The geometric features and material properties of common graft materials have been found to be significantly different to those of the native ACL. The graft size is often chosen regardless of the size of the native ACL, while the material properties are dependent on the source of the graft (Musahl et al., 2022). The ACL has an hourglass shape, with the areas of its bone insertion sites being over three times larger than the midsubstance (Wang et al., 2022). In contrast, tendon grafts typically have a relatively constant cross-sectional area (Han and Lee, 2019; Ayanoglu et al., 2022), termed a “columnar” shape in this study. Such differences may result in different mechanical behaviors. Similarly, studies have reported variations in the elasticity of commonly used ACL grafts (Wilson et al., 1999), which can have a direct effect on the resulting mechanical behavior (Han and Lee, 2019). Snaebjornsson et al. (2017) found that each incremental increase of 0.5 mm in graft diameter reduced the likelihood of needing revision surgery by 86%. Wang et al. (2020b) reported that larger graft diameters could better restore joint stability, allowing the graft to withstand greater forces while reducing stress concentrations on the articular cartilage. However, Orsi et al. (2017) found a potential for graft impingement on the femoral intercondylar notch if the mid-section of the graft was too large, with small differences in graft size leading to a considerable increase in impingement forces. Wang et al. (2020a) reported that more flexible grafts (low stiffness) may induce less bone tunnel enlargement and exhibit lower graft wear by reducing the stress concentrations at the contact area between the tunnel entrance and the graft. These studies indicate that a small diameter on a columnar graft may compromise joint stability and function, while an overly large diameter may lead to impingement. Also, the graft material is critical for proper functionality of the knee joint. Achieving a balance between these factors may be key to improving the longevity and performance of ACL reconstruction. However, it is unclear whether the size and properties of columnar grafts can be effectively tailored to replace the function of the hourglass-shaped native ACL.

As such, using finite element modeling, this study aims to assess 1) whether the diameter and elasticity of columnar grafts can be tailored to fully restore knee joint stability and graft force while avoiding graft impingement, and 2) whether an hourglass-shaped graft or columnar graft can better replicate the function of the native ACL. Finite element analysis (FEA) was used in this study because it allows the variate to be repeatedly changed without damaging the sample and the variate can be strictly controlled to one factor when the other factors are maintained constant. These advantages allow for different diameters, material properties and shapes of grafts used for ACLR to be assessed individually in this study. The outcomes of this study may provide a scientific basis for improving the design of ACL grafts and improving the clinical outcome of ACL reconstruction.

## 2 Materials and methods

### 2.1 Finite element model of the human knee joint

**FIGURE 1:**
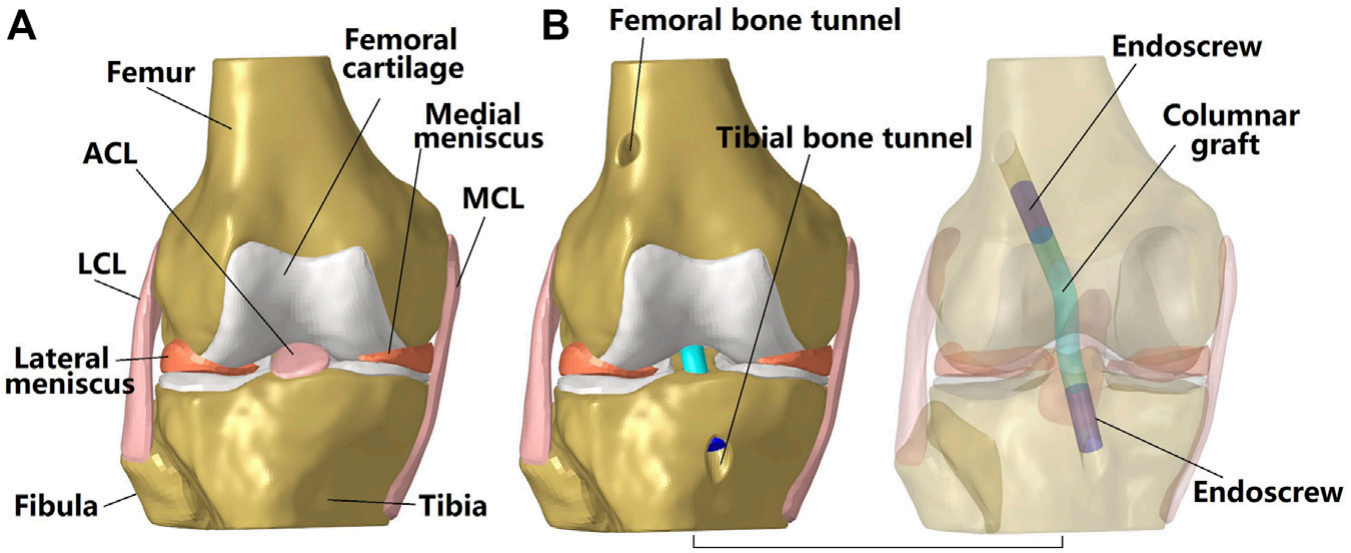
Three dimensional model of a cadaveric knee. **(A)** Intact knee; **(B)** Knee after ACL reconstruction using a columnar graft. The transparent model shows the fixation of the graft to the bone tunnel using an endoscrew. One end of the endoscrew was tied with the graft while the outside surface of the screw was tied with the inside wall of the bone tunnel. LCL, lateral collateral ligament; MCL, medial collateral ligament.

This study used a previously validated finite element (FE) model (Figures 1A, 3A) for all simulations (Wang et al., 2020c). The geometry of the femur, tibia, fibula, articular cartilage, menisci, ACL, posterior cruciate ligament, medial collateral ligament and lateral collateral ligament were segmented from MRI images taken with a scan resolution of 0.2 mm × 0.2 mm and slice thickness of 0.2 mm. The model was meshed in HyperMesh 12.0 (Altair Engineering, Tokyo, Japan) using 4-node tetrahedron elements. A mesh convergence test was used to optimize the element size, whereby a 2.5-mm translational load was applied to the tibia while the femur was fixed at full extension of the knee joint to calculate the *in situ* force in the ACL. The element size was decreased until there was a negligible change in the *in situ* force in the ACL. The resulting element size was 1 mm. The model had a total of 659,251 elements. The material properties of the tissues were defined according to previous literature (Song et al., 2004; Wang et al., 2019). Bones (Young’s modulus = 0.4 GPa, Poisson’s ratio v = 0.33) and cartilage (Young’s modulus = 5 MPa, Poisson’s ratio v = 0.46) were assumed to be linear isotropic elastic tissues. Menisci were assumed to be orthotropic elastic tissues (E_θ_ = 125 MPa, E_R_ = E_Z_ =27.5 MPa, G_θR_ = G_θZ_ = 2 MPa, G_RZ_ = 10.34, V_θR_ = V_θZ_ = 0.1 and V_RZ_ = 0.33). Ligaments were defined as isotropic hyperelastic tissues using strain energy functions. The Veronda-Westmann function was used to define the properties of the ACL and PCL ( α _ACL_ = 0.3 MPa, β _ACL_ = 12.20, α _PCL_ = 0.18 MPa and β _PCL_ = 17.35), while the Mooney-Rivlin material model was used to define the mechanical properties of the MCL and LCL. The coefficients of the LCL were assumed to be identical to those of the MCL (C1 = 30.1 MPa and C2 = −27.1 MPa). The model was validated using data from cadaveric experiments in terms of joint kinematics and ACL forces under an anterior tibial load, valgus tibial moment and internal tibial moment. The model had a calculation accuracy of 0.1 mm for anterior tibial translation, 1 ° for valgus tibial rotation and 1 N for ACL force.

### 2.2 Simulation groups, boundary and loading conditions, and evaluation indicators

The following simulation groups were included in this study: 1) Intact state (group I); 2) ACL reconstruction (ACLR) using a columnar graft with different graft diameters (group II); 3) ACLR using a columnar graft with different graft elasticities (group III); 4) ACLR using an hourglass-shaped graft resembling the dimensions of the native ACL in terms of the cross-sectional area (CSA) of its bone insertion sites and isthmus (group IV).

**FIGURE 2:**
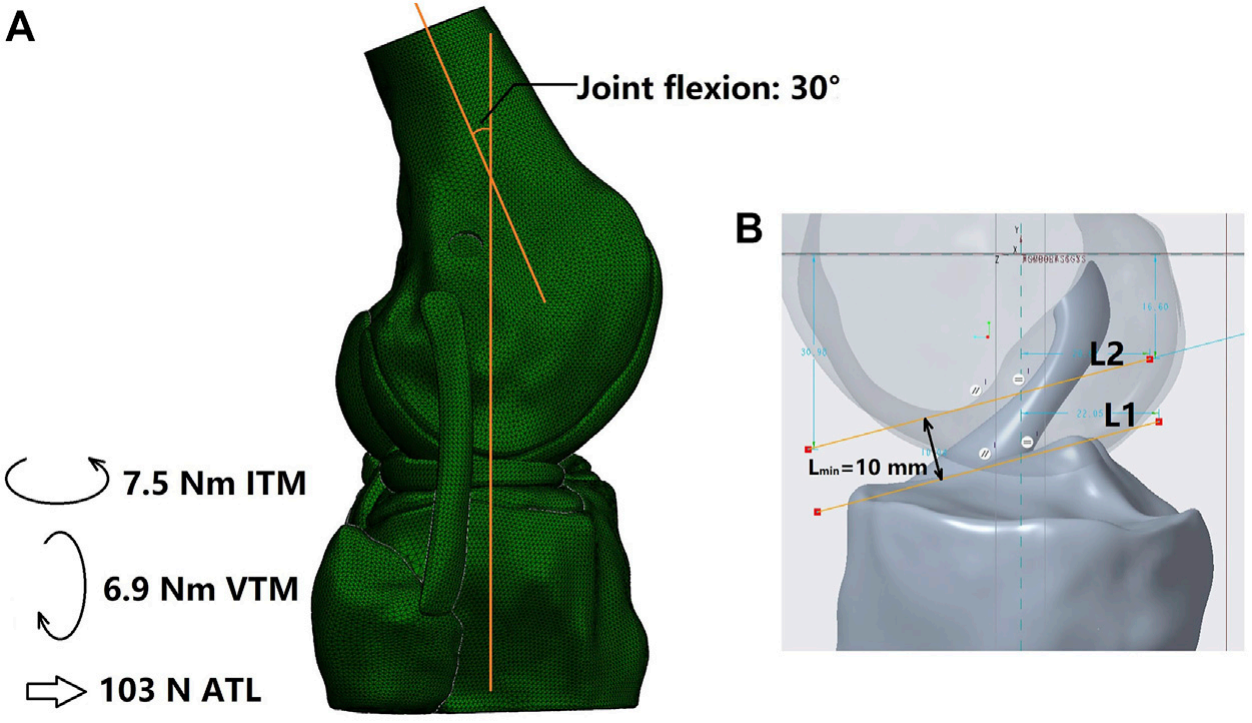
**(A)** Loading conditions and **(B)** Measurement of the minimum width of the space between the femoral notch and tibial plateau (L_min_). L1 was plotted as a line tangent to the tibial plateau and parallel to L2 and L2 was tangent to the top contour of the femoral notch. ITM, internal tibial moment; VTM, valgus tibial moment; ATL, anterior tibial load.

Using the software Abaqus/CAE 6.14–2 (Simulia, Inc., United States), all simulation groups were assigned the same boundary and loading conditions (Figure 2A). First, the tibial shaft was fixed in 6 degrees of freedom (DOFs) and the femur was flexed to 30 ° . Next, the femoral shaft was fixed in six DOFs while the tibial shaft was fixed only in flexion-extension. The bottom surface of the tibial shaft was then subjected to an anterior tibial load of 103 N, internal tibial moment of 7.5 Nm, and valgus tibial moment of 6.9 Nm. A flexion angle of 30 ° was used because a previous study has shown the peak ACL force during normal gait to occur at this joint position. Besides, the ACL was previously reported to be mainly functional for restraining anterior tibial loads and pivot shift loads (internal and valgus tibial moments) (Sakane et al., 1997; Kanamori et al., 2000), thus the combined loads used in this study represented the maximum values experienced during normal gait and were considered as a worst case for tensioning the ACL (Kutzner et al., 2010).

Joint stability under the above loading conditions were recorded to evaluate the stability and stiffness of the knee, including the anterior tibial translation (ATT), internal tibial rotation (ITR) and valgus tibial rotation (VTR). Greater knee displacement than the intact state indicates knee instability in the corresponding DOFs, and less knee movement may indicate over-restraint of the joint. The *in situ* forces in the ACL and grafts were also obtained to evaluate the restoration of normal functionality. A lower force borne by the graft in comparison to the native ACL would indicate insufficient restoration, which may produce greater loading in other joint tissues and increase the risk of secondary damage.

The risk of graft impingement on the femoral notch was evaluated by comparing the effective diameter at the graft isthmus (D) against the minimum width of the space between the femoral notch and the tibial plateau (L_min_) (Figure 2B). For the columnar graft, the diameter was constant along the whole graft, while the D value for the hourglass-shaped graft was calculated by Eq. 1. Then, Eq. 2 was used to calculate the risk for graft impingement on the femoral notch. Higher values for R_ImP_ indicate a greater risk of graft impingement, and a value greater than 1 indicates that the graft cannot be adequately accommodated within the femoral intercondylar notch at full knee extension.

$$\left.D=2\times \sqrt{\frac{S_{isthmus}}{\pi }}\right.$$ (1)

$$R_{ImP}=\frac{D}{L_{\min}}\times 100\%$$ (2)

where D is the effective diameter at the graft isthmus, S_isthmus_ is the cross-sectional area of the graft isthmus, and R_ImP_ is the risk of impingement.

The results from the ACLR groups (group II, III, IV) were compared with the intact group (group I) to evaluate the effectiveness of each technique at restoring graft and knee function.

### 2.3 ACL reconstruction using columnar grafts with different diameters

This study simulated anatomical single bundle ACL reconstruction (Figure 1B). The centers of the femoral and tibial bone insertion sites were set as the entrances for the femoral and tibial bone tunnels respectively. The angles between the femoral tunnel axis and the horizontal and sagittal planes were 45 ° and 25 ° , respectively, and the angles between the tibial tunnel axis and the horizontal and sagittal planes were 65 ° and 25 ° , respectively (Wan et al., 2012). The columnar grafts were modeled as cylindrical bodies using Creo Parametric 7.0 (Parametric Technology Corporation, United States) to simulate the geometry of an auto-hamstring tendon graft or an artificial graft. The Endoscrew was similarly modeled as a cylinder of length 10 mm and with the same diameter as the graft to fix the graft to the bone tunnel (Hung et al., 2014). One end of the endoscrew was tied with the end of the graft and its exterior wall was tied with the bone tunnel wall to simulate a secure fixation (no relative motion was allowed between the tied contact surfaces). The length of the graft inside the femoral tunnel was 20 mm and inside of the tibial tunnel was 10 mm due to a shorter tunnel. The length of the graft inside the femoral tunnel was decreased by 10 mm to explore the effect of fixation position on the main outcomes of this study. The endoscrew had a Young’s modulus of 110 GPa and Poison’s ratio of 0.35 to simulate a Titanium material.

**FIGURE 3:**
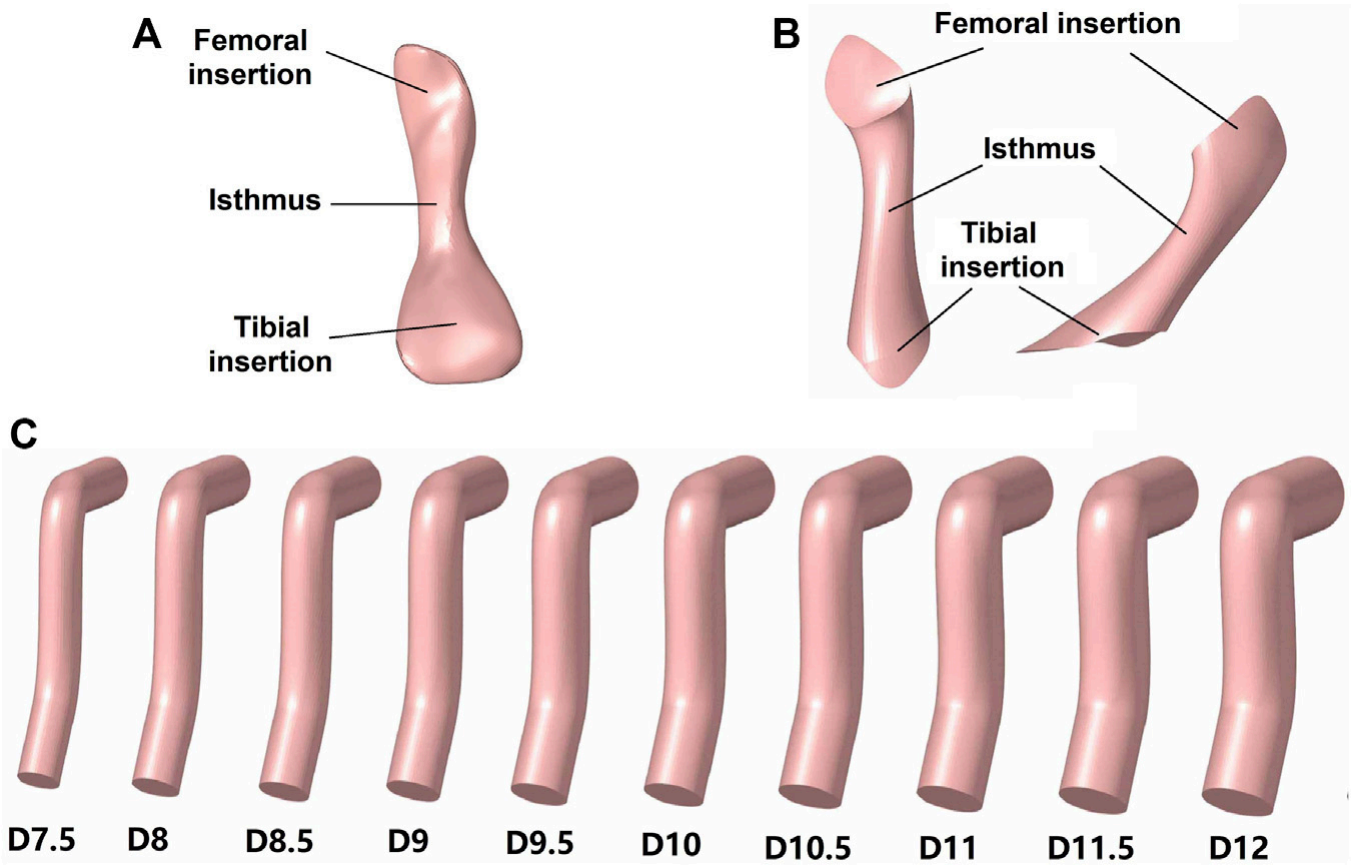
Models of the ACL and grafts. **(A)** Native ACL; **(B)** Hourglass-shaped ACL; **(C)** Columnar grafts with different diameters (D).

For the simulation group II, different graft diameters were evaluated, but the material properties were constant across all models (Young’s moduli = 129.4 MPa), thus to simulate different diameters of a certain type of graft tendon (e.g., hamstring tendon with a diameter of 8 mm vs. 10 mm). Pujol et al. (2013) reported that the effective diameter of an auto-hamstring tendon graft is approximately 10% larger than the native ACL, and, accordingly, this study considered graft diameters ranging from 7.5 to 12 mm, increasing in increments of 0.5 mm (Figure 3C). The diameter of the bone tunnels was the same as the graft diameter used.

### 2.4 ACL reconstruction using columnar grafts with different material elasticity

Previous studies reported a wide variation in the elasticity of commonly used materials for ACL reconstruction (Butler et al., 1984; Wilson et al., 1999; Smeets et al., 2017; Chivot et al., 2020). For groups III and IV, the grafts were modeled with three different Young’s moduli (129.4, 168.0, and 362.2 MPa) (Butler et al., 1984; Wilson et al., 1999) and a Poisson’s ratio of 0.45 to assess whether the material properties of columnar grafts can be effectively tailored to replicate the functionality of the native ACL. All values for the Young’s moduli were obtained from literature. The Young’s modulus of 129.4 MPa was sourced from a previous experimental study on a subject of a similar age to the current study (Wilson et al., 1999), simulating an auto-graft source. And the moduli of 168.0 MPa (Wilson et al., 1999) and 362.2 MPa (Butler et al., 1984) were obtained from anatomical data from younger people, which may be considered as the properties of allografts obtained from younger tissue donors.

### 2.5 ACL reconstruction using hourglass-shaped grafts

Group IV used an hourglass-shaped graft to replace the native ACL in the knee joint (Figure 3B). The graft model was built in Creo Parametric 7.0 (Parametric Technology Corporation, United States) using mixed scanning whereby three sections of the graft (proximal, isthmus and distal sections) were defined and then connected by a smooth wrapped surface. The proximal and distal sections were centered on the bone insertion sites, and the isthmus was aligned with the native ACL. The cross-sectional area of the three sections replicated those of the native ACL (121.3, 35.3, and 185.6 mm^2^, respectively). The normal lines of the proximal and distal sections were parallel to the tunnel axes in group II and the normal line of the isthmus section was parallel to the cross-sectional plane of the native ACL isthmus.

## 3 Results

### 3.1 Evaluation of risk of notch impingement

**FIGURE 4:**
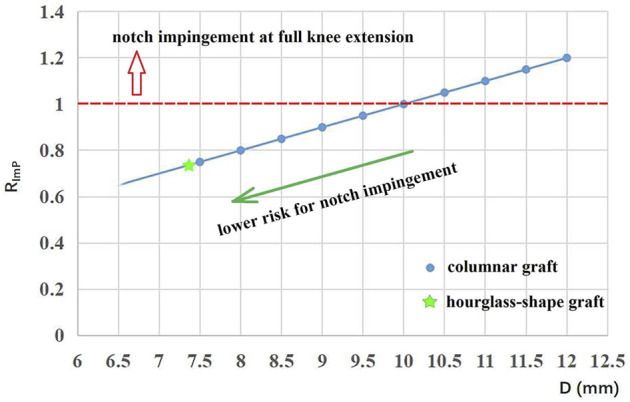
Plot of R_ImP_ for columnar grafts with different diameters and the hourglass-shaped graft.

The minimum height between the femoral notch and tibial plateau was measured as 10 mm. The corresponding values of R_ImP_ for the columnar and hourglass-shaped grafts are shown in Figure 4. The red dotted line represents a value of 1 for R_ImP_. Grafts with data points lying at and above the red dotted line (columnar grafts with a diameter of 10–12 mm) could not be adequately accommodated within the femoral intercondylar notch at full knee extension, and thus were at higher risk of impingement (R_imp_ = 100%–120%). Grafts with data points below the red dotted line (columnar grafts with a diameter of 7.5–9.5 mm, and the hourglass-shaped graft) were at lower risk of notch impingement (R_imp_ = 75%–95%), and the risk decreased as the diameter was reduced. The hourglass-shaped graft had a lower risk of notch impingement than all of the columnar grafts (R_imp_ = 74%).

### 3.2 Evaluation of knee stability and graft force using columnar grafts with different diameters

Knee stability in different DOFs and ligament force for groups I and II are shown in Table 1. Compared with the intact knee (group I), ACLR using a columnar graft with a diameter of 7.5 mm resulted in increased anterior tibial translation (ATT) (4.2 mm vs. 4.7 mm) and valgus tibial rotation (VTR) (0.9 ° vs. 1.3 ° ), but a reduced *in situ* force in the graft (128 N vs. 111 N). However, internal tibial rotation (ITR) was consistent with the intact knee (12.1 ° ). Increasing the graft diameter resulted in a reduction in ATT, ITR and VTR, but lead to greater force on the graft. None of the columnar grafts simulated were capable of replicating both the stability and ligament force of the intact knee at the knee flexion angle of 30°. Although the results from the knee with a 11.5 mm graft could be considered most similar to the intact knee, the ITR was still over-restrained (12.1 ° vs. 11.0 ° ) and the VTR was under-restrained (0.9 ° vs. 1.1 ° ).

**TABLE 1:** ATT, Anterior tibial translation; IRT, internal tibial rotation; VTR, valgus tibial rotation and ACL/graft force in the intact knee and knee after ACLR using columnar grafts of different diameters.

		ATT (mm)	ITR (°)	VTR (°)	ACL/graft force (N)
Intact knee with the native ACL		4.2	12.1	0.9	128
Diameter of columnar graft in ACLR (mm) (Young’s modulus of the graft = 129.4 MPa)	7.5	4.7	12.1	1.3	111
	8	4.5	11.9	1.3	113
	8.5	4.5	11.8	1.3	116
	9	4.5	11.7	1.2	117
	9.5	4.4	11.6	1.2	119
	10	4.4	11.5	1.2	119
	10.5	4.3	11.3	1.2	122
	11	4.3	11.2	1.2	124
	11.5	4.2	11.0	1.1	129
	12	4.0	10.8	1.0	133

### 3.3 Evaluation of knee stability and graft force using columnar grafts with different elasticity

As shown in Table 2, increasing the Young’s modulus of the graft resulted in a reduction in ATT, ITR and VTR and an increased force in the graft. The results also indicate that smaller diameter grafts are needed to maintain physiological knee stability and graft force when stiffer materials (high Young’s modulus) are used for the graft. A graft with a Young’s modulus of 168.0 MPa required a diameter of 9 mm to fully restore ATT in line with the intact knee at the knee flexion angle of 30°, which was 2.5 mm smaller than the graft diameter needed for a material of lower Young’s modulus (129.4 MPa). With a larger Young’s modulus of 362.2 MPa, the knee was over-restrained in ATT even with a relatively small graft diameter of 7.5 mm. The ITR for all ACLR knees was all over-restrained regardless of graft diameter or Young’s modulus, except for when using the most flexible graft (129.4 MPa) with the smallest diameter (7.5 mm).

**TABLE 2:** ATT, Anterior tibial translation; IRT, internal tibial rotation; VTR, valgus tibial rotation and ACL/graft force in the intact knee and knee after ACLR using columnar grafts with different elasticity.

		ATT (mm)	ITR (°)	VTR(°)	ACL/graft force (N)
Intact knee with the native ACL		4.2	12.1	0.9	128
Diameter of columnar graft in ACLR (mm) (Young’s modulus of the graft = 129.4 MPa)	7.5	4.7	12.1	1.3	111
	8	4.5	11.9	1.3	113
	8.5	4.5	11.8	1.3	116
	9	4.5	11.7	1.2	117
	9.5	4.4	11.6	1.2	119
	10	4.4	11.5	1.2	119
	10.5	4.3	11.3	1.2	122
	11	4.3	11.2	1.2	124
	11.5	4.2	11.0	1.1	129
	12	4.0	10.8	1.0	133
Diameter of columnar graft in ACL reconstruction (mm) (Young’s modulus of the graft = 168.0 MPa)	7.5	4.4	11.8	1.3	115
	8	4.3	11.6	1.2	116
	8.5	4.3	11.5	1.2	120
	9	4.2	11.4	1.2	122
	9.5	4.2	11.3	1.2	124
	10	4.2	11.2	1.1	127
	10.5	4.1	11.0	1.1	129
	11	4.1	10.9	1.1	134
	11.5	4.0	10.7	1.0	139
	12	3.8	10.4	0.9	147
Diameter of columnar graft in ACL reconstruction (mm) (Young’s modulus of the graft = 362.2 MPa)	7.5	3.8	11.2	1.1	127
	8	3.6	11.0	1.0	132
	8.5	3.6	10.8	1.0	139
	9	3.6	10.6	0.9	142
	9.5	3.6	10.5	0.9	147
	10	3.6	10.3	0.9	152
	10.5	3.6	10.0	0.8	157

### 3.4 Evaluation of knee stability and graft force using hourglass-shaped grafts with different elasticity

As shown in Table 3, an hourglass-shaped graft resembling the dimensions of the native ACL with a Young’s modulus of 129.4 MPa could restore joint stability and graft force in line with the intact knee at the knee flexion angle of 30°. Similar to the columnar grafts, increasing the Young’s modulus of the hourglass graft lead to a reduction in ATT, ITR and VTR, but an increase in graft force. Using stiffer grafts with a higher Young’s modulus (168.0 and 362.2 MPa) caused the knee to be over-constrained in all DOFs assessed, and also lead to a considerable increase in force on the graft in comparison to the intact knee.

**TABLE 3:** ATT, Anterior tibial translation; IRT, internal tibial rotation; VTR, valgus tibial rotation and ACL/graft force in the intact knee and knee after ACLR using an hourglass-shaped graft with different elasticity.

	ATT (mm)	ITR (°)	VTR(°)	ACL/graft force (N)
Intact knee with the native ACL	4.2	12.1	0.9	128
ACLR using an Hourglass-shaped graft with a Young’s modulus of 129.4 MPa	4.2	11.7	0.9	129
ACLR using an Hourglass-shaped graft with a Young’s modulus of 168.0 MPa	4.1	11.6	0.8	142
ACLR using an Hourglass-shaped graft with a Young’s modulus of 362.2 MPa	4.1	11.3	0.5	169

### 3.5 Comparing the restoration of knee stability and graft force between the conventional columnar graft and hourglass-shaped graft with the same elasticity

**FIGURE 5:**
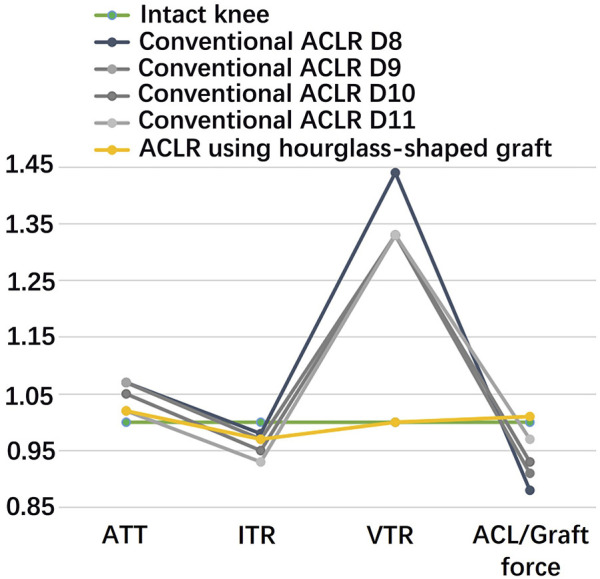
Comparison of a conventional columnar graft to an hourglass-shaped graft for restoring knee stability and ligament force. The values were standardized against an intact knee. D8 signifies a graft diameter of 8 mm. ATT, anterior tibial translation; ITR, internal tibial rotation; VTR, valgus tibial rotation.

Figure 5 shows that with grafts of the same elasticity (Young’s modulus = 129.4 MPa), the hourglass-shaped graft was better able to simultaneously restore the knee stability and graft force to a level resembling the intact knee at the knee flexion angle of 30°. In contrast, the conventional columnar graft with varying diameters resulted in a lax ATT and VTR, but with the ITR over-constrained and a graft force lower than the intact ACL.

### 3.6 Effect of the position for graft fixation inside the bone tunnel on the main outcomes

**FIGURE 6:**
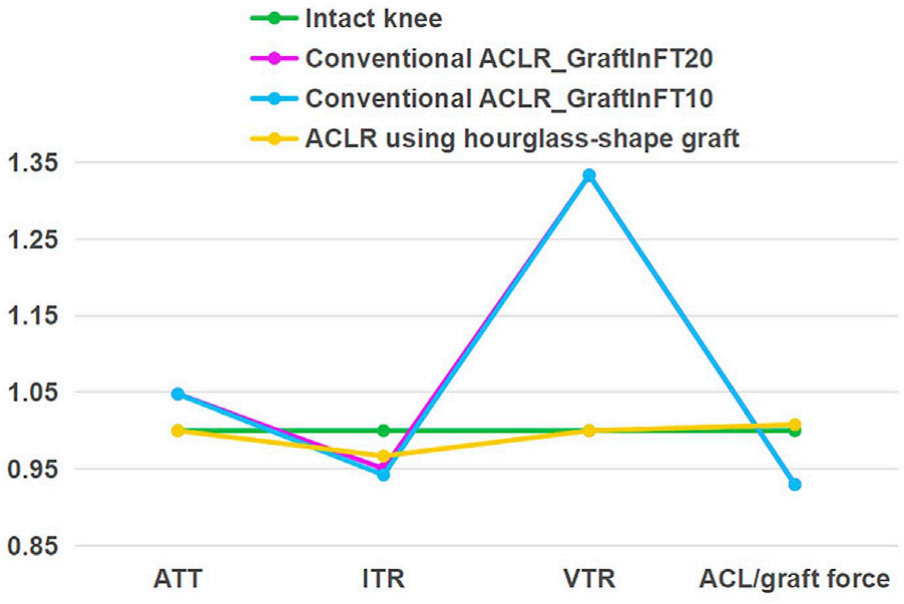
Comparison of a conventional columnar graft (diameter 10 mm, Young’s moduli = 129.4 MPa) to an hourglass-shaped graft for restoring knee stability and ligament force, with varying position of graft fixation in the femoral tunnel (10 mm vs. 20 mm) for the conventional ACLR. GraftInFT20 means the graft length inside the femoral tunnel was 20 mm. The values were standardized against an intact knee. ATT, anterior tibial translation; ITR, internal tibial rotation; VTR, valgus tibial rotation.

As shown in Figure 6, comparing with the simulation group using a graft length of 20 mm inside the bone tunnel, a shorter graft length (10 mm) inside the bone tunnel caused less ITR, when the ATT, VTR and graft force were not changed. Changing the length of the graft inside the bone tunnel did not change the outcome that comparing with the conventional ACLR group, the hourglass-shaped graft was better able to simultaneously restore the knee stability and graft force at the knee flexion angle of 30°.

## 4 Discussion

This study found that the diameter and elasticity of columnar grafts in ACLR could not be tailored to simultaneously replicate the knee stability and graft force of an intact knee at the knee flexion angle of 30°. Although increasing the diameter and/or Young’s modulus of the graft could improve knee stability and graft force, it was not possible to restore both the joint stability and graft force at the same time. Larger diameter columnar grafts were also found to be at risk of impingement with the femoral notch. In contrast, an hourglass-shaped graft resembling the dimensions of the native ACL was better able to simultaneously restore knee stability and graft function at the knee flexion angle of 30°, while avoiding graft impingement, and thus may be at lower risk of rupture, instability and over-restraint of the knee.

In comparison to the intact knee, the results showed an increase in ATT and VTR after ACLR using a columnar graft of diameter 7.5 mm with a Young’s modulus of 129.4 MPa. Notably, a graft diameter of 7.5 mm is commonly used in clinical practice when using an auto-hamstring tendon graft (Pujol et al., 2013). Higher values of ATT and VTR represented greater knee instability than the intact knee, which may cause an abnormal stress environment on the articular cartilage and, in the long term, may lead to knee OA. Meanwhile, the graft force was lower than the force in the native ACL, indicating the graft may not be capable of withstanding physiological joint forces at the knee flexion angle of 30°. This may impart larger forces on other tissues in the knee joint, such as the menisci or articular cartilage. Increasing the graft diameter improved knee stability and increased the force in the graft, indicating that choosing a suitable graft diameter is critical for restoring knee kinematics and forces in the tissues (Snaebjornsson et al., 2017; Wang et al., 2020b). However, this study found that columnar grafts could not replicate both the knee stability and ligament force of the intact joint at the same time. It was possible to restore one parameter to a physiological level, but at the expense of the other. For example, ATT was restored to the level of the intact joint only when ITR was over-restrained, indicating that the knee joint after conventional ACLR might be in the state of instability in one DOF while being over-restrained in another. When the knee moves in a different way to the intact knee, this may change the magnitude and distribution of articular stress (Wang et al., 2020b), which may cause degeneration of the cartilage in the long term (Arokoski et al., 2000). The results also showed that graft diameters larger than 10 mm are at high risk of impingement with the intercondylar femoral notch. This may be problematic when the data suggests that a graft diameter larger than 10 mm is needed to adequately restore knee functionality to a physiological level. Similarly, Howell et al. (1991) found that most columnar grafts suffer impingement on the femoral notch if the bone tunnels are placed at the centers of the bone insertion sites of the native ACL, and thus roofplasty or posterior placement of the tibial tunnel are often required to prevent such impingement. Unfortunately, from the author’s point of view, placing the bone tunnels away from the native bone insertion sites may result in abnormal joint movement and damage to the knee. Notchplasty is also commonly performed by the surgeons to avoid the impingement. However, notchplasty disrupts the normal structure of the knee and may introduce other unforeseen complications. In this case, use of an hourglass-shaped graft may be helpful for avoiding these issues.

Increasing the Young’s modulus of the graft was shown to restrict joint movement and increase the graft force, which could be expected to improve knee stability and reduce the risk of secondary damage to the other joint tissues. Beveridge et al. (2019) reported that 90% of the variability in gross cartilage changes (measured by macroscopic scoring) was associated with ACL stiffness (obtained by *ex vivo* tensile testing) at 6 months after ACL repair, with a higher ACL stiffness producing less cartilage damage (Beveridge et al., 2019). This finding is supported by the results of this current study, where an increase in graft elasticity was shown to improve knee stability and graft force, and thus may improve the articular stress and reduce damage to the cartilage. Also, increasing the Young’s modulus allowed for a smaller graft diameter to be used while maintaining similar levels of knee stability and graft force, meaning less tissue needs to be harvested from the donor site. However, regardless of the Young’s modulus or diameter, this study found that columnar grafts were not able to simultaneously restore both knee function and graft force to a near-physiological level at the knee flexion angle of 30°.

In contrast, using an hourglass-shaped graft resembling the dimensions of the native ACL may allow for the graft isthmus to be more easily accommodated by the femoral notch. This may reduce the risk of graft impingement after ACLR. The hourglass-shaped graft was also able to simultaneously restore both knee stability and graft force to levels resembling the intact knee at the knee flexion angle of 30°. The graft was developed by measuring the area of the native bone insertion sites and ACL isthmus and then blending these areas to create a smooth graft model. This development method is both accurate and easy to replicate, making it suitable for clinical applications in cases where the bone insertion sites and ACL isthmus can be measured *in vivo*. It should be noted that increasing the Young’s modulus of the hourglass graft restricted knee movement and increased graft force, indicating that choosing suitable mechanical properties based on the required graft size is critical for the success of ACLR surgery. Also, given that an hourglass-shaped graft may be difficult to obtain directly from human tissue, allografts or artificial grafts may be a more suitable way for applying this method to clinical practice.

This study has some limitations. 1) The Young’s moduli of the grafts were obtained from literature, but the reported properties of grafts in literature can vary considerably and so the moduli used in this study may be different from those used in clinical practice. As shown by this study, larger graft elasticity resulted in lower knee laxity and greater graft force. This study suggests that the mechanical properties of ACL grafts should be carefully evaluated and selected for ACLR to adequately restore knee functionality. 2) All grafts were considered to have isotropic material properties. However, in clinical practice, the graft properties is anisotropic and can undergo minor changes during implantation due to its viscoelasticity. Nevertheless, the main function of the ACL is to bear tensile forces and the loading conditions used in this study primarily act to tension the ACL and grafts. Assigning isotropic properties to the ligaments should have little effect on the main findings of this study. The models in this study did not consider graft viscoelasticity, which may result in a stiffer joint than a physical knee. As such, future studies may further explore the effect of viscoelasticity on graft functionality after ACLR. 3) The internal tibial rotation (ITR) may be over-constrained in this FE model in comparison to the native ACL because the longitudinal fibers of the ligament were not considered, and thus the rotations of fibers could not be simulated. This may result in a lower simulated ITR of the knee. 4) Only a static loading condition was considered. Effectiveness of using the hourglass-shaped graft may vary in different knee flexions and different loadings such as dynamic loadings representing different daily activities. Future studies should further evaluate its effectiveness under more complex loading environments. 5) Different fixation techniques may lead to variation in the stiffness of the bone-graft-bone complex. In the current study, a good fixation between the graft and bone tunnel was assumed, which permits no relative motion between them. Such a completely rigid fixation neglected the graft sliding along the longitudinal axis of the bone tunnels and may result in a lower knee laxity after ACLR than the real situation.

In the current clinical practice, auto-grafts are often more convenient to use and can reduce the risk of immune reactions, but they can also compromise knee functionality, as shown in the current study. The currently used graft size is usually depending on the used tendon. We thus look forward to future developments of advanced biotechniques for better processing allografts and biomaterials to produce more effective artificial grafts for better restoring the joint functionality.

## 5 Conclusion

At the knee flexion angle of 30°, the hourglass-shaped graft was better able to restore joint stability and graft force after ACLR than columnar grafts. The hourglass graft might also be beneficial for reducing the risk of impingement on the femoral notch. The findings of this study provide information on designing and choosing suitable grafts depending on the conditions of the native knee, and thus may improve the clinical outcome of ACLR.

This study provides innovative concepts for designing and choosing suitable grafts for ACLR, especially when using allografts or artificial grafts, and thus may improve the clinical outcome of ACLR.

## Data Availability

The original contributions presented in the study are included in the article/Supplementary Material, further inquiries can be directed to the corresponding author.
